# Evaluating an Integrated APS–Elder Advocate Intervention Model in the State of Maine

**DOI:** 10.1093/geroni/igaa057.2436

**Published:** 2020-12-16

**Authors:** David Burnes, Marie-Therese Connolly, Patricia Kimball, Stuart Lewis, Erin Salvo

**Affiliations:** 1 University of Toronto, Toronto, Ontario, Canada; 2 University of Southern California Leonard Davis School of Gerontology, Washington, District of Columbia, United States; 3 Elder Abuse Institute Of Maine, Brunswick, Maine, United States; 4 Hofstra/Northwell Health, New York City, New York, United States; 5 Maine Adult Protective Services, Augusta, Maine, United States

## Abstract

Despite recommendations to include a distinct intervention phase in Adult Protective Services (APS), most APS programs close cases following investigation/substantiation phases without engaging in a defined intervention phase. This study implements and evaluates a novel APS service planning/intervention model in the state of Maine. Using an experimental efficacy trial design with stratified random sampling at the level of Maine APS offices, this study compares standard APS care with an enhanced/integrated APS intervention model involving “elder advocates”. Advocates were trained in motivational interviewing, supported decision-making, teaming, restorative justice, and goal attainment scaling to develop capacity to work with both the older adult victim and perpetrator and to strengthen the family and social systems surrounding the victim-perpetrator dyad. This presentation will present results on the efficacy of this integrated APS/elder advocate model and discuss the challenges and successes in conducting elder abuse intervention research in collaboration with APS and APS clients. Part of a symposium sponsored by Abuse, Neglect and Exploitation of Elderly People Interest Group.

